# P63 Spectrum of respiratory bacterial and viral pathogens in individuals with and without influenza at the Emirates Hospital Group, Dubai, UAE

**DOI:** 10.1093/jacamr/dlaf230.070

**Published:** 2025-12-04

**Authors:** Francesca Cainelli, Mohamad Chamas, Wajiha Amin, Afraa Abdulrahim Abdulrahman Abdullah, Ghada Moustafa Abdellatif Ibrahim, Sushma Uttam Kanukale, Shiji Thampy, Hussain Nasser Saleh Alrahma, Nayzak Tahir Raoof, Wael Sammur

**Affiliations:** Emirates Hospital Jumeirah, Dubai, UAE; Emirates Specialty Hospital, Dubai, UAE; Emirates Hospital Jumeirah, Dubai, UAE; Emirates Specialty Hospital, Dubai, UAE; Emirates Hospital Jumeirah, Dubai, UAE; Emirates Hospital Jumeirah, Dubai, UAE; Emirates Specialty Hospital, Dubai, UAE; Emirates Specialty Hospital, Dubai, UAE; Emirates Specialty Hospital, Dubai, UAE; Emirates Hospital Jumeirah, Dubai, UAE; Emirates Specialty Hospital, Dubai, UAE; Emirates Hospital Jumeirah, Dubai, UAE

## Abstract

**Background:**

The understanding of the spectrum of respiratory bacterial and viral pathogens in influenza-positive and influenza-negative patients is limited. It is important to identify the pathogens co-responsible for respiratory infections in patients with influenza.

**Objectives:**

To review retrospectively comprehensive respiratory panels performed at Emirates Hospital Group facilities in Dubai from 1st February to 31st July 2025.

**Patients and methods:**

A total of 3033 oropharyngeal swabs were collected from influenza-like cases, defined as fever (body temperature >38℃) with cough and/or sore throat. Total nucleic acid was extracted using the automated extraction MultNAT system, UStar Biotechnologies, Hangzhou. Real-time PCR was performed for three bacteria (*Chlamydia pneumoniae*, *Streptococcus pneumoniae*, *Mycoplasma pneumoniae*) and 20 respiratory viruses (Adenovirus B, Adenovirus C, Bocavirus, Coronavirus 229E, Coronavirus HKU1, Coronavirus NL63, Coronavirus OC43, Enterovirus, Metapneumovirus, Influenza A, Influenza H3N2, Influenza H1N1, Influenza B, Parainfluenza I, Parainfluenza II, Parainfluenza III, Parainfluenza IV, Respiratory Syncytial Virus, Rhinovirus, SARS-Cov 2).

**Results:**

A total of 328 cases (181 males; mean age 12 years, median 4.6) were influenza-positive and 2705 (1512 males; mean age 7, median 3.0) negatives. All viruses and bacteria tested were found in both patient populations. Parainfluenza III (5.0% versus 3.0%, *P*=0.05), Rhinovirus (15.5% versus 6.7%, *P*=0.000028), SARS-CoV-2 (8.5%versus 5.0%, *P*=0.03) were all more frequently detected in influenza-negative patients. Parainfluenza IV was more frequently found in influenza-positive patients (5.0% versus 2.0%, *P*=0.017). A significant proportion of patients (756 cases, including both influenza-positive and negatives) were identified as having mixed infections; 512 were cases of dual infections, 178 of triple infections, 53 of quadruple infections, 8 of quintuple infections and 5 cases of sextuple infections. The most frequent coinfections involved *Streptococcus pneumoniae*, Rhinovirus and Influenza virus. The most common coinfections in influenza-negative patients were Respiratory Syncytial Virus-Rhinovirus (17 cases, 0.6%), Parainfluenza I-SARS-CoV-2 (5 cases, 0.2%), Respiratory Syncytial Virus-SARS-CoV-2 (5 cases) and Respiratory Syncytial Virus-Bocavirus (5 cases).

**Conclusions:**

A high prevalence of respiratory coinfections was found in a prevalently paediatric population presenting to the facilities of a hospital group in Dubai, including in patients with influenza virus infection. Unfortunately, *Haemophilus influenzae* and *Staphylococcus aureus*, frequently associated with influenza-related complications, were not tested. It is important to consider the possibility of not only bacterial but also viral coinfections in patients with influenza virus infections.

